# The Role of the Location of Personal Exposimeters on the Human Body in Their Use for Assessing Exposure to the Electromagnetic Field in the Radiofrequency Range 98–2450 MHz and Compliance Analysis: Evaluation by Virtual Measurements

**DOI:** 10.1155/2015/272460

**Published:** 2015-03-24

**Authors:** Krzysztof Gryz, Patryk Zradziński, Jolanta Karpowicz

**Affiliations:** Laboratory of Electromagnetic Hazards, Central Institute for Labour Protection, National Research Institute (CIOP-PIB), Ulica Czerniakowska 16, 00-701 Warszawa, Poland

## Abstract

The use of radiofrequency (98–2450 MHz range) personal exposimeters to measure the electric field (*E*-field) in far-field exposure conditions was modelled numerically using human body model Gustav and finite integration technique software. Calculations with 256 models of exposure scenarios show that the human body has a significant influence on the results of measurements using a single body-worn exposimeter in various locations near the body ((from −96 to +133)%, measurement errors with respect to the unperturbed *E*-field value). When an exposure assessment involves the exposure limitations provided for the strength of an unperturbed *E*-field. To improve the application of exposimeters in compliance tests, such discrepancies in the results of measurements by a body-worn exposimeter may be compensated by using of a correction factor applied to the measurement results or alternatively to the exposure limit values. The location of a single exposimeter on the waist to the back side of the human body or on the front of the chest reduces the range of exposure assessments uncertainty (covering various exposure conditions). However, still the uncertainty of exposure assessments using a single exposimeter remains significantly higher than the assessment of the unperturbed *E*-field using spot measurements.

## 1. Introduction

These days environmental electromagnetic fields (EMF) are present everywhere and the entire population is exposed. The most common technologies producing radiofrequency EMF exposure to the general public and workers are analogue and digital radio and television broadcasting, mobile communications system for closed groups (such as the TETRA system (terrestrial trunked radio)), the digital cellular network system GSM 900 (Global System for Mobile Communications), the digital cellular network system DCS 1800 (digital communication system), the digital enhanced cordless telecommunications of short distance (DECT system), the digital cellular network system UMTS 2100 (Universal Mobile Telecommunications System), wireless local area networks, for example, access to internet WiFi (wireless fidelity), and broadband data transmission WiMAX (worldwide interoperability for microwave access), and 4G LTE (long-term evolution) [1]. At the same time, EMF at other frequencies are present worldwide, including mainly power frequency magnetic field exposure from outdoor and indoor supplying electric installations.

## 2. EMF Exposure Assessment Strategies

International guidelines regarding the limitations of environmental exposure to EMF focus on preventing acute thermal effects caused by radiofrequency (RF) EMF and the effects of neuroexcitation caused by low frequency fields [2, 3]. The exposure metric related to the thermal load of an exposed body is the “specific energy absorption rate” (recognised as SAR and expressed in W/kg), which denotes how much energy per unit time and unit mass is absorbed by the tissue. Based on the Maxwell equations, SAR is proportional to the square of the electric field (*E*-field, which is an electric component of EMF) induced in tissues (~*E*_in_^2^), which is a function of the external unperturbed electric field (*E* ^2^), but is also dependent on exposure conditions, such as EMF frequency, the spatial distribution of the incident *E*-field, the grounding conditions of the exposed person, and any metal objects nearby. These parameters may characterise a dynamic picture of how the EMF are distributed within the tissue, as well as the level of human body exposure during environmental measurements.

Exposure assessment in epidemiological studies is highly dependent upon the type of disease studied. For instance, the field strengths may be useful measures to obtain in studies focused on effects depending on short-term exposure (i.e., effects of an acute character). In the case of diseases with long latencies, such as cancer or Alzheimer's, it becomes much more difficult, and since then an exposure that occurred at least several years ago is under question. The challenge is to assess exposure retrospectively with reasonable accuracy, covering many influencing factors, such as various types of EMF emitters used in the vicinity of the living/working place of investigated person or varying intensity of activities near EMF sources, for example, the frequency of visiting city centres, where radiofrequency exposure may be many times higher than in the suburbs. Moreover, the question of if and how exposure is accumulated over the years is still under scientific debate [1, 4]. Therefore, in studies of the health effects of EMF exposure, the assessment of the exposure pattern over a longer time-scale is (or should be) also an important task, for example, to evaluate the parameter of exposure over days or even years, recognised as the “dose.” “Dose” concept in exposure assessment assumes a time-dependence of effect, for instance, due to the accumulation of cell/tissue damage [1, 4]. In the process of developing the international safety guidelines a tissue heating caused by EMF exposure plays the role of the “established effect”^1^ considered as the rationale for the limits regarding exposure to radiofrequency EMF. “Dose” concept is not relevant in this case, since there is no known accumulation of thermal damage below exposure limits. However, in epidemiological studies, where long-term effects of repeated or continuous exposure over years are studied, the determination of an EMF “dose” is one of the main tasks (even though the concept of “dose” has not been well-developed yet, and various protocols for evaluating it may be found in the literature). This is mainly because the interaction mechanisms relevant to long-term exposure effects, especially where weak fields are concerned, are not well understood, meaning that the question of the relevant metrics of exposure is still open [1, 4].

Both low frequency magnetic fields and radiofrequency EMF have been classified by IARC as “a possible carcinogenic to humans,” category 2B [1, 5]. As a result, radiofrequency EMF exposure may be evaluated as the main environmental factor for the studied health end-point or as a cofactor affecting all studied end-points. This option of the combined effects of exposure is strengthened by recently published papers on genotoxic effects in vitro and in vivo after a single exposure to combined fields from magnetic resonance scanners (including static magnetic fields, intermediate frequency magnetic field, and radiofrequency EMF) [6, 7]. The cumulative measures of EMF exposure (the “dose”) have been already used in epidemiological studies regarding health effects of exposure to low frequency and radiofrequency EMF, for example, the dose defined as “*μ*T·years” in low frequency studies. In addition, other metrics of exposure were used in epidemiological studies, for instance, in studies on the relation between mobile phone use and brain tumours, the total number of calls, or the total accumulated call time [8, 9] and, more recently, the total energy accumulated in the tumour [10, 11]; however, the biological relevance of these measures is still not clear.

## 3. Environmental EMF Exposure Limitations

Because of harmful health effects (taking long-term and short-term effects into account) exposure to RF-EMF (radiofrequency electromagnetic field) is limited in relation to workers and the general public. Council of the European Union Recommendation 1999/519/EC provides limits for general public exposure to EMF in the range from 0 Hz to 300 GHz [12]. This recommendation is nonbinding and the rules of general public protection against EMF are not unified over European countries. Some countries have adopted limits directly from Recommendation 1999/519/EC to national law (e.g., Cyprus, Czech Republic, Estonia, Finland, France, Hungary, Ireland, Malta, Portugal, Romania, and Spain [13]). Other countries use stricter limits (e.g., Belgium, Bulgaria, Greece, Italy, Lithuania, Luxembourg, Poland, Slovenia, and the autonomous Spanish region of Catalonia).

In the area of occupational exposure, Directive 2013/35/EU on the minimum health and safety requirements regarding the exposure of workers to risks arising from electromagnetic fields (EMF Directive) is under binding transposition to the national law of European countries [14]. However, each country may decide to establish more restrictive limits of workers' exposure than in the directive. Examples of exposure limits for workers or the general public to RF-EMF are given in Table 1.

In European documents as well as in national legislation and international guidelines and standards, exposure limits were defined for unperturbed EMF, that is, without a human presence in the evaluated space.

## 4. EMF Exposure Assessment Tools

For an assessment of the hazards caused by acute exposure effects, measurements of the actual exposure are relevant. However, when studying the effects of chronic exposure and the relationship to diseases such as cancer, a personal measurement of exposure over the time of activities in exposed locations is welcome, in order to help in the evaluation of the pattern of exposure in the time scale. Changes in the exposure level over time may be caused by the exposed subject moving or by changes in the emitted power from the EMF source. It is also important to note that the interaction of the fields and the body will depend on the exact shape and position of the exposed individual, as well as the frequency and polarisation of the incident EMF.

In the case of exposure to complex EMF from many sources, identifying the frequency of the dominant component of exposure or the frequency composition of exposure is of great interest. Available devices allow measurements of both the actual values of the electric (*E*, in V/m) and magnetic (*H*, in A/m) field strength of unperturbed EMF, as well as sampling the *E* and *H* values over time. Small battery-powered data loggers of *E* or *H* values, recognised as “exposimeters,” may be used in various ways, for measuring the actual value of the unperturbed field, for measuring the actual value of the field at the body surface or for measuring the variability of both kinds of fields over time, with a sampling rate defined by the user. The use of body-worn exposimeters allows the pattern of individual exposure to EMF to be measured (monitored) over the time scale and its variability caused by the activity of the individual person to be followed.

The most widely used RF-EMF or more precisely *E*-field exposimeters are dedicated for investigations of hazards caused by various wireless telecommunication systems from the frequency band (88–5850) MHz, existing in working and living environments. Exposimetric technique of measuring in the frequency selective mode allows more detailed RF-EMF investigations in comparison to the classic measurement technique, involving broadband measurements of electric or magnetic field strengths in selected locations (i.e., spot measurements, which covers both components of exposure together, far-field, and near-field). The frequency response of frequency-selective exposimeters corresponds to the frequency of RF-EMF sources currently in use in the public environment:

- FM radio broadcasting: (88–108) MHz,
- television broadcasting: TV3 (174–223) MHz and TV3&4 (470–830) MHz bands,
- mobile communications system for closed groups: TETRA (380–400) MHz,
- digital cellular network system GSM 900: with transmission from handsets to base transmitter station (880–915) MHz [known as uplink (UL)] and transmission from base transmitter station to handsets (925–960) MHz [known as downlink (DL)],
- digital cellular network system DCS 1800: (1710–1785) MHz [UL] and (1805–1880) MHz [DL],
- digital enhanced cordless telecommunications of short distance DECT system: (1880–1900) MHz,
- digital cellular network system UMTS 2100: (1920–1980) MHz [UL] and (2110–2170) MHz [DL],
- wireless local area networks, for example, access to the internet: (2400–2500) MHz [WiFi 2G] and (5150–5850) MHz) [WiFi 5G],
- broadband data transmission: (3400–3800) MHz [WiMAX] and (800–2600) MHz [different bands of 4G LTE].

The metrological and technical parameters of the most popular frequency-selective RF-EMF exposimeters are given in Table 2.

Exposimeters are being used in studies regarding the assessment of EMF environmental impact as relatively new research tools. The applicability of this method is still increasing and developing. This exposure assessment method should be limited to assessing the far-field exposure components, because an assessment of near-field exposure needs an evaluation of SAR. Additionally, the conditions of measurements with exposimeters are different in comparison with classic methods, when measurements are performed without the presence of anybody in the assessed EMF, and measurement results are compared with limit values provided by international safety guidelines or standards [2, 3, 12, 14]. It must also be pointed out that when exposimeters are worn on the body, the results of measurements may be far from the unperturbed field, which should be assessed when compliance with mentioned exposure limits is assessed. In the case of using body-worn exposimeters, the measurements of the unperturbed *E*-field are not available. The results of exposimetric measurements depend on many factors, including the influence of the human body on the EMF spatial distribution. This is caused by the absorption and reflection of EMF, which are frequency-dependent because they depend on the dielectric properties and the relative dimensions of the body to the wavelength of incident EMF (Table 3).

Frequency selective exposimeters allow RF-EMF to be measured simultaneously in each frequency band and for the level of exposure to be correlated with sources, which may help to split far-field components of exposure from the near-field components, as well as to evaluate the uncertainty component in measurement results coming from the influence of the human body on the incident EMF spatial distribution. Manufacturers' data do not usually cover detailed information on the variations between the unperturbed EMF (assessed by classical measurements) and EMF measured by body-worn exposimeters.

## 5. The Aim of the Work

The aim of the work was to carry out numerical modelling of the use of RF-EMF exposimeters (a kind of virtual measurements) regarding various measurement protocols, including various locations of the exposimeters on the body, as well as the frequency, propagation, and polarisation of the measured *E*-field. The obtained results of the modelling were analysed to assess the influence of the human body on the RF-EMF measurement results when personal exposimeters are used in various ways. Finally, there was an analysis of the possibility of optimising the position of the exposimeter for assessing the compliance of EMF exposure with the exposure limits of workers or the general public, which were set for unperturbed field evaluation.

The available experience with the use of RF-EMF frequency selective exposimeters is focused on evaluating general public exposure in epidemiological studies. The research presented in this paper was performed with the intention of improving the ability to properly use exposimeters also in the area of compliance tests, regarding the exposure limits of workers or the general public.

## 6. Material and Methods

**Figure 1:**
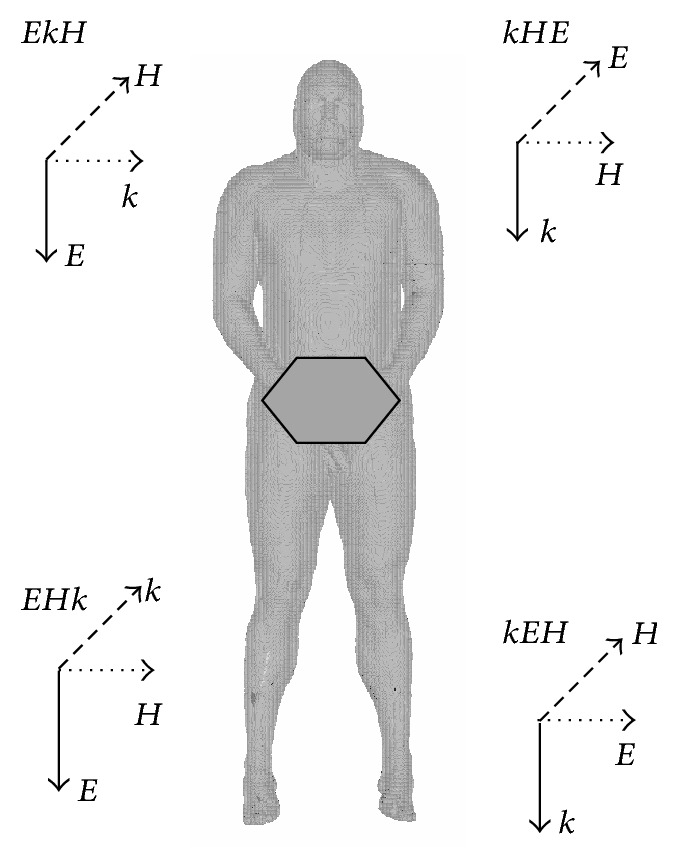
Directions of RF-EMF propagation (*k*), *E*-field vector polarisation (*E*), and *H*-field vector polarisation (*H*) in relation to the Gustav model.

Numerical calculations were performed for four exposure scenarios corresponding to typical exposure conditions in the environment, characterised by various directions of RF-EMF propagation (*k*) and *E*-field vector polarisation (*E*) in relation to the Gustav model (Figure 1). The three-letter coding of exposure scenarios used in later sections of this paper follows WHO publications [15], with letter-order corresponding to field components polarisation or direction of field propagation in regard to vertical (top, bottom), transverse (right, left), or sagittal (front, back) axis of the human body. The investigated exposure scenarios are given and described in Table 4.

Calculations were taken for EMF frequencies and the dielectric properties of tissues relevant for the central frequency in bands of the frequency-selective exposimeters (corresponding to typical RF applications which cause exposure in far-field conditions): FM radio, 98 MHz, TV3, 200 MHz, TETRA, 390 MHz, TV4&5, 650 MHz, GSM(DL), 943 MHz, DCS(DL), 1843 MHz, UMTS(DL), 2140, and WiFi, 2450 MHz. They covered frequencies of wireless telecommunications systems existing almost everywhere in the environment. Taken together, they are the dominant source of far-field exposure of the population.

Simulations were carried out using the anatomical male body model Gustav (height 186 cm, weight 69 kg, and spatial resolution 2.08 × 2.08 × 8 mm, composed of 57 tissues) placed in a free space with boundaries at a distance of 1 m from each side (horizontally) and 1.5 m from the top of the model (vertically). All the boundaries were set to open (add space) which extends the calculation domain of 1/2 wavelength (i.e., in the investigated frequency range, 0.06–1.5 m). The Gustav model was insulated from the ground (located at a height of 1/2 wavelength above the bottom boundary).

Only exposure to EMF from sources located at a distance from the user of RF-EMF exposimeters was modelled, where an exposure assessment may be performed based on the *E*-field measurements, because this may be counted as far-field exposure. In the case of using mobile phone handsets very close to the user's body, for example, the exposure assessment needs to be based on the other method, SAR calculations.

Numerical models were composed of 15–18 million voxels, with the smallest voxel dimensions inside the human body model of approximately 2 mm, complying with minimum requirements of the resolution of body models used for SAR calculations, 1/10 of the wavelength in human model tissues [16, 17]. For the investigated frequencies, the minimum resolution is approximately 20 mm for (98 MHz); 4 mm for (943 MHz); and 2 mm for (2450 MHz).

The uncertainty of the numerical simulations carried out, including components of uncertainty related to model resolution, numerical method, dielectric parameter of tissues of used human body model, field source, and voxels accuracy, was evaluated as ±(32–41)%. This is in line with the uncertainty of ±41% reported in the standard IEC 62232:2011 as “rationale uncertainty” of such simulations in the frequency range 300 MHz–6 GHz [16]. The amplitude of the continuous wave (CW) *E*-field on the boundary of space emitting RF-EMF (EMF source model) was constant at 10 V/m.

The methodology for performing measurements with the use of exposimeters is not standardised. Usually exposimeters are located in different ways, in order to ensure that they do not impede the normal activity of the exposed person. The manufacturer's data for ESM-140 exposimeters advises that they be mounted on the arm. Others exposimeters did not have any information regarding the advised location of the exposimeter on the body of the human whose exposure is under consideration.

**Figure 2:**
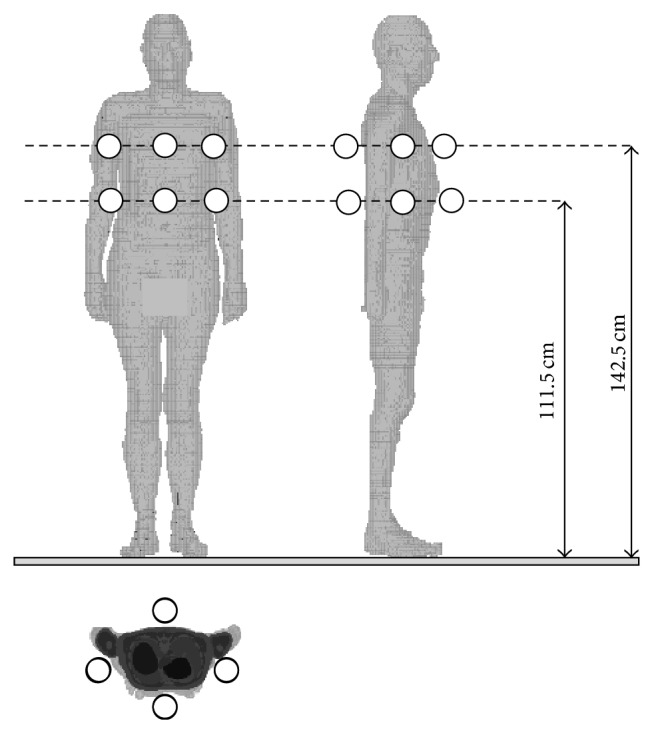
Locations of point-assessments of *E*-field: view from the front, right side of the Gustav model and from the top to cross-section of the Gustav model.

On the basis of the calculation results, the *E*-field at points located 111.5 cm and 142.7 cm height (corresponding to the height of the waist and chest of the Gustav model) at a distance of 5 cm (typical distance from the centre of the measurement sensor of the exposimeters to the body surface) from the front (F), back (B), left (L, shadowed by the body between exposimeter and the EMF source), and right (R, directly exposed by EMF from the source) side of the Gustav model were established. These locations of point-assessments of the *E*-field represented the location of the geometrical centre of the exposimeter *E*-field sensor mounted on the human body (at the waist or the chest), the virtual model of measurements (Figure 2).

**Figure 3:**
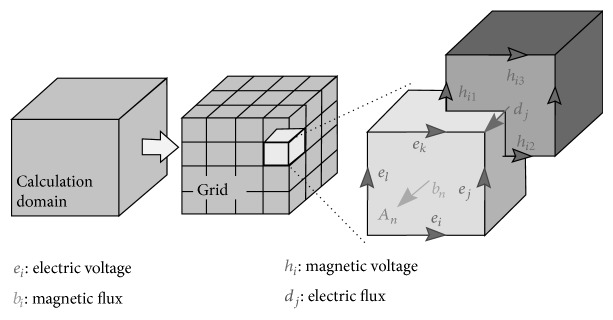
Discretisation in the FIT method with electric and magnetic components [27].

Investigations were based on numerical modelling using the CST Studio Suite 2012 software, with a package dedicated for high frequencies (microwave) based on the finite integration technique [27]. CST Studio Suite 2012 is a general-purpose electromagnetic simulator (used in many electromagnetic problems) based on the integral form of Maxwell's equations. To solve these equations numerically, a finite calculation domain is defined and split up into many small elements or grid cells (Figure 3).

Maxwell's equations are formulated for each of the cell facets separately. Considering Faraday's Law, the closed integral on the equation's left side can be rewritten as the sum of four grid voltages without introducing any supplementary errors. Consequently, the time derivative of the magnetic flux defined on the enclosed primary cell facet represents the right-hand side of the equation. By repeating this procedure for all available cell facets matrix, a sufficient representation of the analysed problem is obtained. Doing it in the same way with other of Maxwell's equations and using Ampère's law in the case of a dual grid, allows a complete discretised set of Maxwell's grid equations (MGEs) to be obtained. Taking into account the material parameters for each cell, all matrix equations are available to solve EMF problems for the discrete grid space.

The obtained results of *E*-field strength were analysed by calculating the statistical parameters of numerical calculations using the software package Statistica, Version 10.0PL (StatSoft, USA). For each exposure scenario (coded following Table 4 and Figure 1), the analysis covered minimum, maximum, and average value of parameter *D*, defined according to the formula

(1) $$\begin{array}{c} D=100\%\frac{E-E_{u}}{E_{u}}, \end{array}$$

where *E* is electric field strength calculated at a defined measurement point close to the human body model (as presented at Figure 2), equivalent to the result of measurement by a body-worn *E*-field meter, and *E*_*u*_ is unperturbed electric field strength calculated at the same measurement point, in the absence of any human presence.

Positive values of the *D* parameter mean an overestimation, and negative *D* values mean an underestimation of the exposure level when using a single exposimeter.

The influence of the body on the results of measurements using an exposimeter at a particular location, calculated for various RF-EMF directions of propagation and *E*-field polarisation, represents various positions of the person equipped with an exposimeter against the EMF source (e.g., turning around the body axis).

## 7. Results

**Figure 4:**
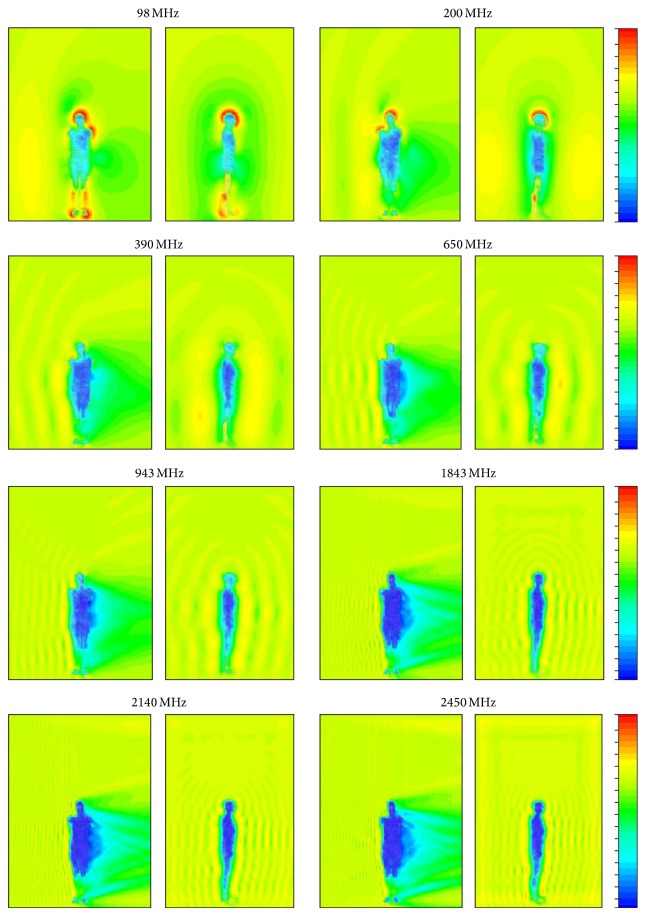
An example of the cross-section of spatial distribution of the *E*-field in exposure scenario coded* EkH* for frequencies 98, 200, 390, 650, 943, 1843, 2140, and 2450 MHz, standardised linear scale of colours.

**Figure 5:**
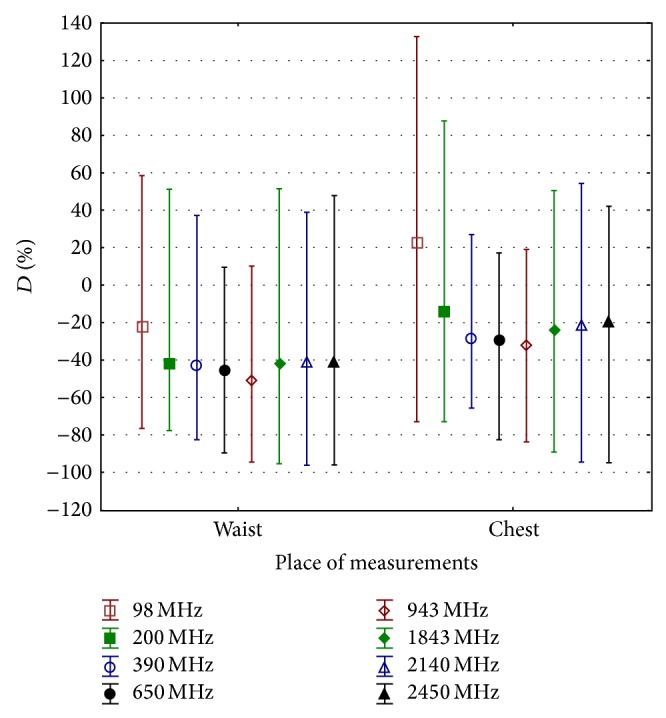
The difference between the unperturbed *E*-field and the results of exposimetric measurements in various locations near the human body, at the waist and on the chest (parameter *D*); at particular frequencies, each subgroup of 32 results for various exposure scenarios in all directions of RF-EMF propagation and *E*-field vector polarisations (defined in Table 4) and 4 locations is characterised by the range from minimum to maximum values (represented by whiskers) and average value (represented by a square).

**Figure 6:**
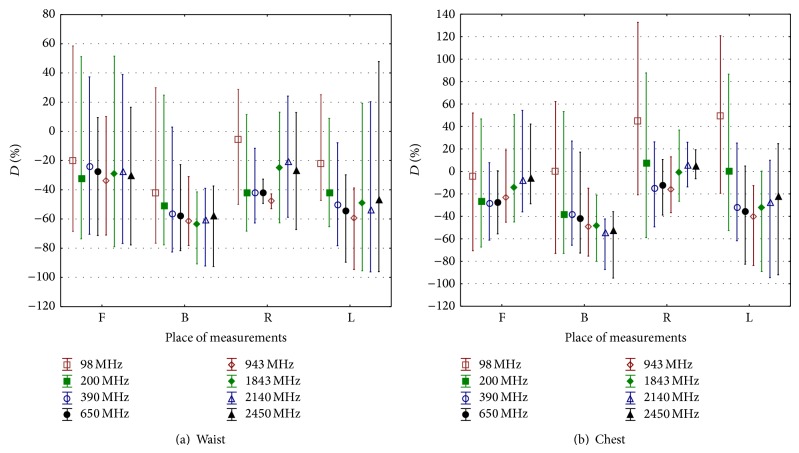
The difference between the unperturbed *E*-field and results of exposimetric measurements in a different location near to the human body (parameter *D*): F, front, B, back, L, left side, and R, right side; at particular frequencies, each subgroup of four results for various exposure scenarios in all directions of RF-EMF propagation and *E*-field vector polarisations (defined in Table 4) is characterised by the range from minimum to maximum values (represented by whiskers) and average value (represented by a square).

An example of the spatial distribution of the *E*-field in 256 analysed virtual models is shown in Figure 4 regarding the exposure scenario coded* EkH*. An example of statistical analysis (minimum, maximum, and average value) of the *D* parameter calculated in subgroups of analysed scenarios is shown in Figure 5 (regarding results split into subgroups defined by the waist and chest location of the measurement point and the EMF frequency) and Figure 6 (regarding results split into subgroups defined by the location of the exposimeter).

The results of 256 virtual measurements show that the *E*-field measured near the body (i.e., by exposimeters) significantly differs in comparison with the unperturbed field (i.e., the *E*-field measured without any human presence in the vicinity of the measurement device), in various exposure scenarios and various protocols of using a single exposimeter: *D* = (from −96 to +133)% over the frequency range 98–2450 MHz. Exposimeter locations near the waist give measurement results less influenced by the human body than locations near the chest: *D* = (from −96 to +58)% [average value *D*_aver_ = −41%] and *D* = (from −95 to +132)% [average value *D*_aver_ = −18%], respectively (Figure 5).

## 8. Discussion

The values of the *D* parameter represent the influence that the proximity of the human body has on the *E*-field spatial distribution. These values need to be analysed when exposimetric measurements are used in compliance tests regarding the exposure limits. In the present environmental exposure limitations, the assessment requires an evaluation of unperturbed fields. Because of that, the proper correction factor to compensate for shadow and reflection influence on exposimetric measurement results needs to be applied when such results are compared with the criteria given for the unperturbed field.

The values of the *D* parameter were calculated with respect to the *E*-field unperturbed value at the location of the exposimeter; however, because of the simulation of exposure to the homogeneous field, they may also be considered with regard to the whole-body averaged *E*-field.

The results obtained in our study by virtual measurements are compatible with laboratory investigations and numerical calculations presented in the literature [18–21]. Bolte et al. investigated the influence of the body on the results of measurements using an exposimeter located at the waist (at 108 cm height) of a male body for the horizontal and vertical polarisation of the *E*-field and for various positions of the person (turning around the body axis) [18]. For the vertical polarisation of the *E*-field and all frequency bands of the exposimeter, the reported values of the *D* parameter were from the range (from −96 to +151)% (e.g., for a particular bands FM, GSM(DL), and WiFi 2G it was (from −38 to  −73)%, (from −90 to +35)%, and (from −93 to +1)%, resp.). The results of measurements regarding the *E*-field in the frequency range 100–5200 MHz, performed using an exposimeter worn on the body (at the waist), as presented by Loader et al., were on average 50% lower in relation to the measurement results of the unperturbed field inside an anechoic environment [19].

In numerical simulations using an anatomical male model (the visible human phantom (VHP), similar to that used in the presented study), for the location of the exposimeter on the hips, arms, and back, the obtained values of the *D* parameter calculated with regard to the whole-body averaged *E*-field were (from −70 to +186)%, (from −91 to +70)%, (from −93 to +84)%, and (from −92 to +105)%, for frequencies 100, 946, 2140, and 2450 MHz, respectively [20]. In other numerical simulations using the anatomical model of the male human body Norman and a single plane wave using various exposure scenarios at frequency 900 MHz and in 30 random locations of measurement points in front of the torso, at distances of 1 and 5 cm from the body surface, it has been shown that the average underestimation of the *E*-field value can reach −52% [21].

The discrepancies observed in the results of virtual measurements between exposimetric measurement results and the values of the unperturbed *E*-field are caused by shielding or reflecting effects in EMF propagation near the body and depend on the relative configuration of the exposimeter, the human body, and the RF-EMF source. In the presented study, as well as in the mentioned simulations using the VHP model [20], the highest overestimation was found at 98 MHz frequency (especially for RF EMF propagation along the axis of the body). This may be the result of EMF energy absorption by the human body, which happens most strongly in the frequency range neighbouring the resonant frequency for the EMF absorption of electromagnetic energy (i.e., 40–80 MHz, where the equivalent electric height of the human body is close to 1/4 of the EMF wavelength). The highest underestimation was found for 943 MHz.

The averaged value of parameter *D* obtained from the reported study (i.e., corresponding to particular subgroups of virtual measurements) is in the range *D* = (from −20 to  −50)% (unipolar influence) in models representing the use of the exposimeter near the waist and in EMF of various frequencies, while the use of the exposimeter near the chest gives *D* = (from −30 to +20)% (bipolar influence). Given that, it is easier to compensate for the unipolar influence on the exposimetric measurement caused by the body in the case of the waist exposimeter position than the bipolar influence in the chest position. The distribution of the *D* parameter presented at Figure 5 shows that it may be expected that a 40% underestimation of exposure level is most typical, and when carrying out the exposure evaluation against exposure limits (set for an unperturbed *E*-field), it may be compensated by

- the correction factor of *A*1 = 0.6 (applied to the exposure limit values),
- the correction factor of *A*2 = 1.7 (alternatively applied to the exposimetric measurement results).

The use of such correction factors, reflecting the characteristic of a measurement session, may be complicated in general because exposure scenarios are usually dynamically changing as the exposimeter user is allowed to move around the EMF source or is present in the space of multipath propagation of EMF from outlying sources. However, in some cases, the location of the worker's activity is sufficiently fixed to allow for the reasonable use of these correction factors. Discrepancies in the *E*-field exposure assessment through the use of an RF body-worn exposimeter are also strongly dependent on the location of the exposimeter. Taking into account the distribution of *D* parameter values in subgroups of results representing exposimeter results at various exposimeter locations and human body movement against the EMF source location, the lowest range of *D* values were obtained for the exposimeter location on the front of chest (Figure 6). Next location minimizing values of *D* parameter is at the waist from the back side of the human body. Such locations of exposimeter reduce the range of uncertainty in results of exposimetric measurements between various exposure scenarios. They are also relatively convenient for the person who is carrying exposimeter during the measurement.

To summarise, because of the changes in the values of such correction factors, corresponding to various exposure scenarios, the uncertainty of the exposure assessment through the use of a single body-worn exposimeter remains significantly higher than the assessment of the *E*-field based on spot measurements of the unperturbed field (which is achievable in the range of 15–25% in the case of RF EMF). In light of the presented analysis of the results of virtual measurements modelling the use of single body-worn exposimeter at various locations, a “realistic uncertainty level” of the RF EMF exposure assessment by such an exposimeters should be counted in the range of approximately (from −100 to +150%). However, it must also be pointed out that this uncertainty may be slightly reduced by the use of exposimeter located at the front of the chest (from −65 to +45%) or back of the waist (from −90 to +30%) (Figure 6).

It must also be pointed out that the discussed results disregard practical problems when assessing localised *E*-field exposure, when discrepancies between the results of measurements from a single exposimeter and the values of the unperturbed *E*-field should be counted as higher than presented in this paper. When the distance between the exposed body and the EMF source is large, it may help to use the *E*-field spatially averaged over the whole body or through the use of more than one exposimeter. But at short distances from the source (i.e., a mobile phone handset), international guidelines advise the use of a SAR evaluation instead of *E*-field measurements [2, 3, 12, 14].

## 9. Conclusions

The presented investigations show a significant influence of the human body on the results of RF EMF measurements using body-worn exposimeters. Such measurement errors (with respect to the unperturbed *E*-field value) depend on the frequency of the assessed RF *E*-field and on the location of the exposimeter on the body. A location on the waist from the back side of the human body or in front of the chest limits the range of assessment uncertainty.

In the case of exposure assessments with respect to the exposure limitations provided for the strength of the unperturbed *E*-field, the significant difference between the unperturbed field and the RF exposimeter indications should be taken into consideration. In an environmental test regarding compliance with the binding requirements, the use of a correction factor (applied to the measurement results or alternatively to the exposure limit values) may compensate for such discrepancies between the result of an exposimetric measurement and the exposure metric expected by the exposure limit provider (such as international organization or national legislator). However, because of the influence on the values of such correction factors from exposure configuration, the uncertainty of exposure assessments using a single exposimeter remains significantly higher than the assessment of the *E*-field based on spot measurements of the unperturbed field.

## Figures and Tables

**Table 1:** Examples of RF EMF environmental exposure limits.

Requirements	Frequency *f*, MHz	Electric field strength *E*, V/m
Directive 2013/40/EU, workers [14]	10–400	61
	400–2000	3*f* ^1/2^
	2000–6000	140

Council of the European Union Recommendation 1999/519/EC, general public [12]	10–400	28
	400–2000	1.375*f* ^1/2^
	2000–300000	61

Belgium (Flemish), general public [13]	900	21^(1)^
	1800	29^(1)^
	2100	31^(1)^

Bulgaria, general public [13], workers	900–2100	6.1
		61

Greece, general public [13]	900	32 (35)^(2)^
	1800	45 (49)^(2)^
	2100	47 (51)^(2)^

Italy, general public [22]	3–3000	20^(3)^
		6^(4)^
	3000–300000	40^(3)^
		6^(4)^

Lithuania, general public [13]	900–2100	6.1

Luxembourg, general public [13]	900	41^(5)^
	1800	58^(5)^
	2100	61^(5)^

Poland, general public, workers [23]	3–300000	7
		7–200

Slovenia, general public [13]	900	13^(6)^
	1800	18^(6)^
	2100	19^(6)^

Switzerland, general public [24]	900	4
	1800	6
	900 and 1800	5

Turkey, general public [25]	10–400	7^(7)^
		18^(8)^
	400–2000	0.341*f* ^1/2^ ^(7)^
		1.375*f* ^1/2^ ^(8)^
	2000–6000	15^(7)^
		61^(8)^

*f*, the frequency in MHz.

^(1)^For the broadband spectrum of EMF, from 100 kHz to 300 GHz (except TV and radio transmitters), including mobile phone stations. Per antenna 3.0 V/m (900 MHz), 4.2 V/m (1800 MHz), and 4.5 V/m (2100 MHz).

^(2)^Lower values for or antenna stations closer than 300 m to “sensitive” locations (schools, kindergartens, hospitals, and care homes); elsewhere higher values.

^(3)^Exposure limits, cannot be exceeded under any circumstances.

^(4)^Attention value cannot be exceeded in residential environment.

^(5)^Limit per single device 3.0 V/m.

^(6)^Applied to homes, hospitals, health resorts, public buildings, tourism buildings, schools, nurseries, playgrounds, parks, and recreational areas; otherwise limit for external electric and magnetic field strength equal to reference level in 1999/519/EC.

^(7)^Limit value for a single device.

^(8)^Total limit value for multiple devices.

**Table 2:** Basic parameters of frequency, selective exposimeters (according to manufacturer data).

Parameter	Type of exposimeter, manufacturer			
	ESM-140, Maschek (Germany)	EME SPY 121, Satimo (France)	EME SPY 140, Satimo (France)	EME SPY 200, Satimo (France)
Measurement range (V/m)	0.001–70	0.05–10	0.005–5	0.005–5

Frequency range (MHz)/number of subbands	(880–2500)/8	(88–2400)/12	(88–5850)/14	(88–5850)/20

Sampling rate	0.5–10 s	4–255 s	4–255 s	2-(3-, 4-)255 s^(1)^

Storage capacity (number of memory cells)	260000	12540	80000	80000

Dimensions (mm)	115 × 45 × 29	193 × 96 × 70	169 × 79 × 46	169 × 79 × 50

Weight (g)	87	450	400	440

Marker of events	Yes	Yes	Yes	Yes

Measurement uncertainty	±2 dB/(−20–+25) % (measurement of unperturbed field) ±4 dB/(−36–+58) % (measurement with exposimeters located on the arm)	Not reported	Not reported	Not reported

^(1)^depend on the subband.

**Table 3:** The example of dielectric properties and the wavelength of incident EMF for muscle (transverse fibres) of human.

Frequency, *f*	Conductivity, *σ*	Permittivity, *ε*	Wavelength in tissue (muscle)	Wavelength in air
(MHz)	(S/m)		(m)	(m)
80	0.698	68.8	0.342	3.750
98	0.707	66.2	0.297	3.061
200	0.743	60.2	0.173	1.500
390	0.794	57.2	0.097	0.769
650	0.864	55.8	0.060	0.462
943	0.958	54.9	0.042	0.318
1843	1.365	53.5	0.022	0.163
2140	1.538	53.1	0.019	0.140
2450	1.739	52.7	0.017	0.122
4000	3.016	50.8	0.010	0.075
6000	5.202	48.2	0.007	0.050

Note: conductivity and permittivity following data provided by [26].

**Table 4:** Exposure scenarios used in numerical simulations.

The code of exposure scenario	Direction regarding axis of human body		
	RF-EMF propagation (*k*)	*E*-field vector polarisation (*E*)	*H*-field vector polarisation (*H*)
*EkH *	Transverse	Vertical	Sagittal
*EHk *	Sagittal	Vertical	Transverse
*kHE *	Vertical	Sagittal	Transverse
*kEH *	Vertical	Transverse	Sagittal

Vertical, from top to bottom; sagittal, from front to back; transverse, from right to left.

## Endnotes

1. “Established effects” in International Commission on Non-ionizing Radiation Protection (ICNIRP) approach means the following [2].

- The exposure limitations are intended to protect against diseases and other adverse health effects. Because adverse consequences of EMF exposure can vary from trivial to the life threatening, a balanced judgment is required before deciding on exposure limitation. Biological effects without any identified adverse health consequences do not form a basis for exposure restrictions.
- Because risk assessment is focused on human health, data should ideally be derived from human studies. The relationship between exposure and certain short-term biological effects can sometimes be evaluated from human laboratory studies, whereas data on long-term human effects can only be derived from epidemiological studies. However, epidemiological studies do not provide sufficient evidence of causal relationships without biological data from experimental studies.
- A decision regarding exposure limits must first be made whether available evidence allows the identification of an exposure hazard, that is, an adverse health effect that is caused by EMF exposure. By this identification, the effect becomes established. For a complete risk assessment, selected studies should provide additional, mostly quantitative, data on
  - a definition of a biologically effective quantity, which may vary with organs,
  - an exposure-effect relationship and the identification of a threshold,
  - exposure distribution and the identification of subpopulations with high exposure,
  - differences in a population's susceptibility.
- Reliable risk assessment requires consistent information from multiple studies published in peer-reviewed scientific literature. Therefore, the accumulation of new evidence that may lead to updating health risk assessments is monitored and ICNIRP advice is continuously subjected to revision in light of new evidence, in order to be based on current knowledge.
